# On the Evolutionary History of *Uleiella chilensis*, a Smut Fungus Parasite of *Araucaria araucana* in South America: Uleiellales ord. nov. in Ustilaginomycetes

**DOI:** 10.1371/journal.pone.0147107

**Published:** 2016-01-20

**Authors:** Kai Riess, Max E. Schön, Matthias Lutz, Heinz Butin, Franz Oberwinkler, Sigisfredo Garnica

**Affiliations:** 1 Plant Evolutionary Ecology, Institute of Evolution and Ecology, University of Tübingen, Auf der Morgenstelle 5, 72076, Tübingen, Germany; 2 Am Roten Amte 1 H, 38302, Wolfenbüttel, Germany; National Cancer Institute, UNITED STATES

## Abstract

The evolutionary history, divergence times and phylogenetic relationships of *Uleiella chilensis* (Ustilaginomycotina, smut fungi) associated with *Araucaria araucana* were analysed. DNA sequences from multiple gene regions and morphology were analysed and compared to other members of the Basidiomycota to determine the phylogenetic placement of smut fungi on gymnosperms. Divergence time estimates indicate that the majority of smut fungal orders diversified during the Triassic–Jurassic period. However, the origin and relationships of several orders remain uncertain. The most recent common ancestor between *Uleiella chilensis* and *Violaceomyces palustris* has been dated to the Lower Cretaceous. Comparisons of divergence time estimates between smut fungi and host plants lead to the hypothesis that the early Ustilaginomycotina had a saprobic lifestyle. As there are only two extant species of *Araucaria* in South America, each hosting a unique *Uleiella* species, we suggest that either coevolution or a host shift followed by allopatric speciation are the most likely explanations for the current geographic restriction of *Uleiella* and its low diversity. Phylogenetic and age estimation analyses, ecology, the unusual life-cycle and the peculiar combination of septal and haustorial characteristics support *Uleiella chilensis* as a distinct lineage among the Ustilaginomycotina. Here, we describe a new ustilaginomycetous order, the Uleiellales to accommodate *Uleiella*. Within the Ustilaginomycetes, Uleiellales are sister taxon to the Violaceomycetales.

## Introduction

**Fig 1:**
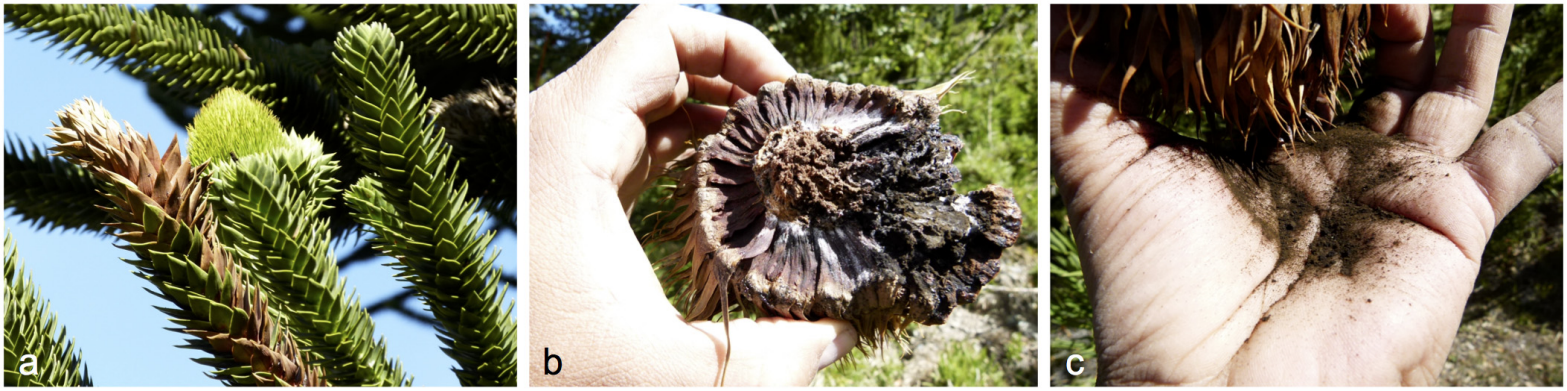
Female cone of *Araucaria araucana* infected by *Uleiella chilensis*. (a) Overview. (b) Dark olive teliospore deposit. (c) Hand with teliospore powder.

With more than 1500 known species, smut fungi (Ustilaginomycotina) represent a highly diverse group of plant parasites [1]. Teliospore-forming species predominantly parasitize non-woody herbs (typically grasses, Poaceae), whereas those without teliospores prefer trees or shrubs [1, 2]. A few species parasitize ferns [3] or conifers [1]. Some species with yeast or yeast-like growth, or with dimorphic life cycles are saprobic [4] or parasitic on animals [5, 6]. Hypotheses on the evolution of smuts have focused either on their origin as parasites of the ancestors of monocot families or an earlier origin, followed by diversification on grass-like monocots [4]. The geographic distribution of plant-parasitizing Ustilaginomycotina has either been interpreted as the result of (i) habitat specializations rather than host preferences [1, 7]; (ii) host jumps to closely or distantly related plant species [2, 4]; or (iii) cospeciation events [8–10]. For instance, *Uleiella* is a unique ustilaginomycotinous genus occurring on gymnosperms restricted to the genus *Araucaria* in South America. There are two species, *Uleiella chilensis* on female cones of *Araucaria araucana* (Fig 1) in Chile and Argentina and *U*. *paradoxa* on male cones of *A*. *angustifolia* in Brazil [11]. The relationship between *Uleiella* and *Araucaria* provides a model to explore the origin and evolution of parasitism. Currently, species of *Araucaria* have disjunct distributions in the Southern Hemisphere, although this genus was widely distributed in both hemispheres during the Mesozoic around 251 to 65 mya [12–15]. Divergence time estimates from the study by Kranitz *et al*. [16] indicated that the stem origin of *Araucaria* was in the Early Cretaceous to Paleocene (~138–60 mya) and that of the Araucariaceae in the Permian–Triassic (~284–202 mya). However, fossils of *Araucaria* were dated as far back as the Lower Jurassic (~ 200 to 176 mya) [17]. It is unclear (i) whether the parasitic association between *Uleiella* and *Araucaria* is the result of co-evolution or a host jump; (ii) whether *Uleiella* predates its host plant; and (iii) whether *Uleiella* is ancestral to the smut fungi. Traditionally, the taxonomic position of the genus *Uleiella* within the Ustilaginomycotina has been uncertain. Limited ultrastructural data has placed it tentatively in the Ustilaginales [1, 2].

In the present study, the evolutionary history of the gymnosperm smut fungus *Uleiella chilensis* on *Araucaria araucana* is inferred by comparison of subcellular and cellular features with those of related taxa and analysis of nuclear DNA sequences. The evolutionary age of *Uleiella chilensis* and its phylogenetic position within the Ustilaginomycotina are resolved in this study. To address the issue against an absence of fossil records for the Ustilaginomycotina, we assembled a dataset comprising 18S, 28S and *rpb*1 sequences from a representative sampling of Basidiomycota that was calibrated at two nodes, including the fossil ancestors of the orders Boletales and Agaricales. The results of this dataset were used as the basis for a secondary calibration of Ustilaginomycotina using a dataset that included 18S, ITS, 28S, *rpb*2 and *EF1α* sequences. To address the second question, we used the same datasets complemented with newly generated subcellular and cellular data to infer the phylogenetic placement of *Uleiella chilensis* within the Ustilaginomycotina.

## Material and Methods

### Ethics statement

The fungal species used in this study were not protected and specimens were traded according to standard international herbaria policy and loan regulations. Additionally, the sampling sites of recently collected specimens were not protected and specific permits for sampling were not required.

### Taxon sampling

Small fragments of female cones of *Araucaria araucana* (Molina) K. Koch infected with *Uleiella chilensis* Dietel & Neger were collected from beside Highway R-955 near Laguna Galletue, 38°40'50.7"S 71°19'12.9"W (leg. H. Butin, 16 March 1985, TUB 020323; microscopy) and fungal material was collected from two different sites along Highway R-89 between Malalcahuello and Lonquimay, 38°25'51.8"S 71°24'36.1"W (leg. S. Garnica and M. A. Jara, 22 March 2012, TUB 020321 and leg. M. A. Jara, 14 March 2014, TUB 020322; molecular analysis), Region IX of the Araucania, Chile. A culture of *Araucaria araucana* TUB 020322 has been deposited in the Leibniz Institute—German Collection of Microorganisms and Cell Cultures (DSMZ) (DSM 100158). Two DNA sequence datasets were compiled (i) to estimate the relative age of the Ustilaginomycotina within Basidiomycota (dataset 1), and (ii) to estimate the relative ages of *Uleiella chilensis* and major lineages within the Ustilaginomycotina (dataset 2). Dataset 1 was sampled from representatives in the main clades of the Agaricomycotina, Pucciniomycotina and Ustilaginomycotina, plus three ascomycetes as outgroup, including sequences from the 18S, 28S and *rpb*1 (exons B–C) genes for all 86 species. Dataset 2 was sampled from representatives of all ustilaginomyceteous fungi and *Colacogloea peniophorae* (Pucciniomycotina) as outgroup for which at least four of 18S, ITS, 28S, *rpb*2 and *EF1α* gene sequences were available from GenBank (24 species). For the species included in datasets 1 and 2, see S1 and S2 Tables, respectively.

### DNA extraction, PCR, sequencing and sequence editing

Fungal genomic DNA was isolated using the InnuPREP Plant DNA Kit (Analytik Jena, Jena, Germany) following the standard protocol. For each sample, fungal material was ground in liquid nitrogen with a plastic pestle, suspended in 400 μL of extraction buffer and incubated for 1 hour at 50°C. PCR primers RPB1-A and RPB1-C were used to target domains A–C of *rpb*1 following the protocol of Matheny et al. [18]. For *rpb*2, regions 5–11 were amplified with the primer combinations RPB2-5F/RPB2-11bR [19], RPB2-5F/bRPB2-7.1 [20] and bRPB2-6F/RPB2-11bR [19, 20] and PCR conditions as described in [20]. The *EF1α* gene was amplified using the primer combinations EF-526F/EF-2218R, EF-526F/EF-ir and Ef-df/EF-2218R (S. Rehner, http://aftol.org/pdfs/EF1primer.pdf) using the protocol of Rehner & Buckley [21]. The internal transcribed spacer (ITS) region of the rDNA including the 5.8S rDNA and the 5'-end of the nuclear large subunit ribosomal DNA (LSU) were amplified using the primer pairs ITS1F/NL4 [22] and LR0R/LR9 (R. Vilgalys lab, http://biology.duke.edu/fungi/mycolab/primers.htm; [23]) following the protocol of Riess et al. [24]. Positive PCRs were purified using ExoSap-IT^®^ (USB Corporation, Cleveland, OH, USA) diluted 1:6. Sequencing of *rpb*1, *rpb*2, *EF1α* and ITS + LSU was carried out using the amplification primers and additional primers as described for *rpb*1 [25], *rpb*2 [26] and LR3R [23] and LR6 [27] for LSU. Cycle sequencing was accomplished using the BigDye Terminator v. 3.1 Cycle Sequencing Kit (Applied Biosystems, Foster City, CA, USA). Sequencing reactions were run through an ABI Prism 3130*xl* Genetic Analyzer (Applied Biosystems). Sequence chromatograms were checked for accuracy and edited using Sequencher v. 4.9 (Gene Codes Corporation, Ann Arbor, MI, USA).

DNA sequences obtained directly from herbarium specimens were compared to the sequences obtained from cultures (see below). The GenBank (http://www.ncbi.nlm.nih.gov) accession numbers for *Uleiella chilensis* sequences are KF061293 (rDNA), KF061319 (*rpb*1), KF061318 (*rpb*2) and KP413031 (*EF1α*).

### Alignments and phylogenetic reconstructions

Sequences of the small (18S) and large subunit (28S) ribosomal DNA of dataset 1 were aligned independently using MAFFT v. 6.935b [28], applying the E-INS-i method [29]. Both alignments were automatically trimmed if more than 60% of all sequences exhibited gaps [30]. The nuclear DNA sequences from the *rpb*1 gene were aligned using DIALIGN-TX [31] and, in the case of *rpb*1, split into two exons. Highly divergent portions and alignment flanks were excluded using trimAl. Subsequently, the amino acid sequences translated from the DNA sequences were subjected to visual adjustments using Se-Al v. 2.0a11 [32]. Autapomorphic insertions and non-coding segments were removed from each alignment. Finally, all three gene alignments were concatenated (the resulting alignment length was 4398 bp, S1 Data). Ribosomal DNA (18S, ITS, 28S) and protein coding (*rpb*2, *EF1α*) sequences in dataset 2 were aligned and concatenated in the same way as described for dataset 1 (the final alignment length was 5767 bp, S2 Data).

For both datasets, maximum likelihood (ML) trees were computed using RAxML v. 8.1.3 [33] with 1000 bootstrap replicates (bootstrap support, BS) [34]. We used the general time-reversible (GTR) substitution model and the CAT approximation to account for heterogeneity along different evolutionary branches. Additional posterior probability nodal support values were determined in a Bayesian phylogenetic Markov chain Monte Carlo (MCMC) search using MrBayes v. 3.2.2 [35] under the GTR model with a gamma-distributed rate variation. Each search comprised two runs of four chains each for 5 × 10^6^ generations sampled every 100 generations with the first 2.5 × 10^5^ generations discarded as burn-in.

### Divergence time estimations

To estimate divergence times of *Uleiella chilensis*, we used the ML trees as starting trees for a MCMC-based time estimation in BEAST v. 1.8.1 [36]. We transformed branch lengths to ages, calibrating two nodes by using fossils [37, 38] for the Basidiomycota (dataset 1) and used the time estimations for the Ustilaginomycotina within this dataset as a time constraint for the analysis of dataset 2. Calibrated clades were monophyletic in the starting trees and constrained as such in BEAST. For both datasets, 50 million generations were evaluated, sampling trees every 1000 generations with a burn-in of 10%. For more information on BEAST settings and the priors used, see S3 Data (Basidiomycota) and S4 Data (Ustilaginomycotina). After checking for convergence with Tracer v. 1.6 [39], consensus trees were calculated and the age estimations plus the highest density probabilities (HDPs) and posterior probabilities for all nodes were reported. We calibrated two nodes in the Basidiomycota dataset using fossil data: (i) the Suillinae (*Suillus pictus*, *Chroogomphus rutilus* and *Gomphidius roseus*) were calibrated based on a fossil of a Pinaceae-associated suilloid ectomycorrhiza (~50 mya) [37]; (ii) the Tricholomatoid clade (represented by *Panellus stipticus* and *Pleurotopsis longinqua*) was calibrated using a fossil *Archaeomarasmius leggetti* (~90 mya) from mid-Cretaceous amber [38]. Subsequently, we used the age estimation of the split between *Ustanciosporium standleyanum* and *Schizonella melanogramma* as well as the root node of the Ustilaginomycotina from the Basidiomycota dataset as secondary calibration points in dataset 2. The 95% HDP range was taken as a prior and the starting tree was calibrated with the mean age estimation as proposed by Forest [40].

### Light and transmission electron microscopy

For the study of germination and the subsequent culture, spores were spread thinly on water agar (WA) and on malt–yeast–peptone agar (MYP) in Petri dishes kept at room temperature. As soon as germlings were produced, a suitable piece of medium (about 10 mm square) was cut out, transferred to a slide and covered with a cover glass. A small droplet of lactophenol with cotton blue, added to the side of the square of medium, fixed and stained the germlings. A culture was deposited in the Herbarium Tubingense culture collection (TUB F 4418). For light microscopy, living material was examined with an Axioplan microscope (Carl Zeiss) using bright field, phase contrast or Nomarski interference contrast optics. For transmission electron microscopy, fungal material from plant infected tissues and cultures were fixed overnight with 2% glutaraldehyde in 0.1 M of a sodium cacodylate buffer (pH 7.2) at room temperature. Following six transfers in 0.1 M of a sodium cacodylate buffer, samples were postfixed in 1% osmium tetroxide in the same buffer for 1 h in the dark, washed in distilled water and stained with 1% aqueous uranyl acetate for 1 h in the dark. After five washes in distilled water, samples were dehydrated in acetone, using 10-min changes at 25%, 50%, 70% and 95% and three times in 100% acetone. Samples were embedded in Spurr's plastic and sectioned with a diamond knife. Serial sections were mounted on formvar-coated single-slot copper grids, stained with lead citrate at room temperature for 5 min, washed with distilled water and studied with a EM 109 transmission electron microscope (Car Zeiss, Oberkochen, Germany) at 80 kV. For teliospore terminology see [41].

### Nomenclature

The electronic version of this article in Portable Document Format (PDF) in a work with an ISSN or ISBN will represent a published work according to the International Code of Nomenclature for algae, fungi and plants and hence the new names contained in the electronic publication of a PLOS article are effectively published under that Code from the electronic edition alone, so there is no longer any need to provide printed copies.

In addition, new names contained in this work have been submitted to MycoBank from where they will be made available to the Global Names Index. The unique MycoBank number can be resolved and the associated information viewed through any standard web browser by appending the MycoBank number contained in this publication to the prefix http://www.mycobank.org/MB. The online version of this work is archived and available from the following digital repositories: PubMed Central, LOCKSS.

## Results

### Age estimations and phylogenetic relationships

**Fig 2:**
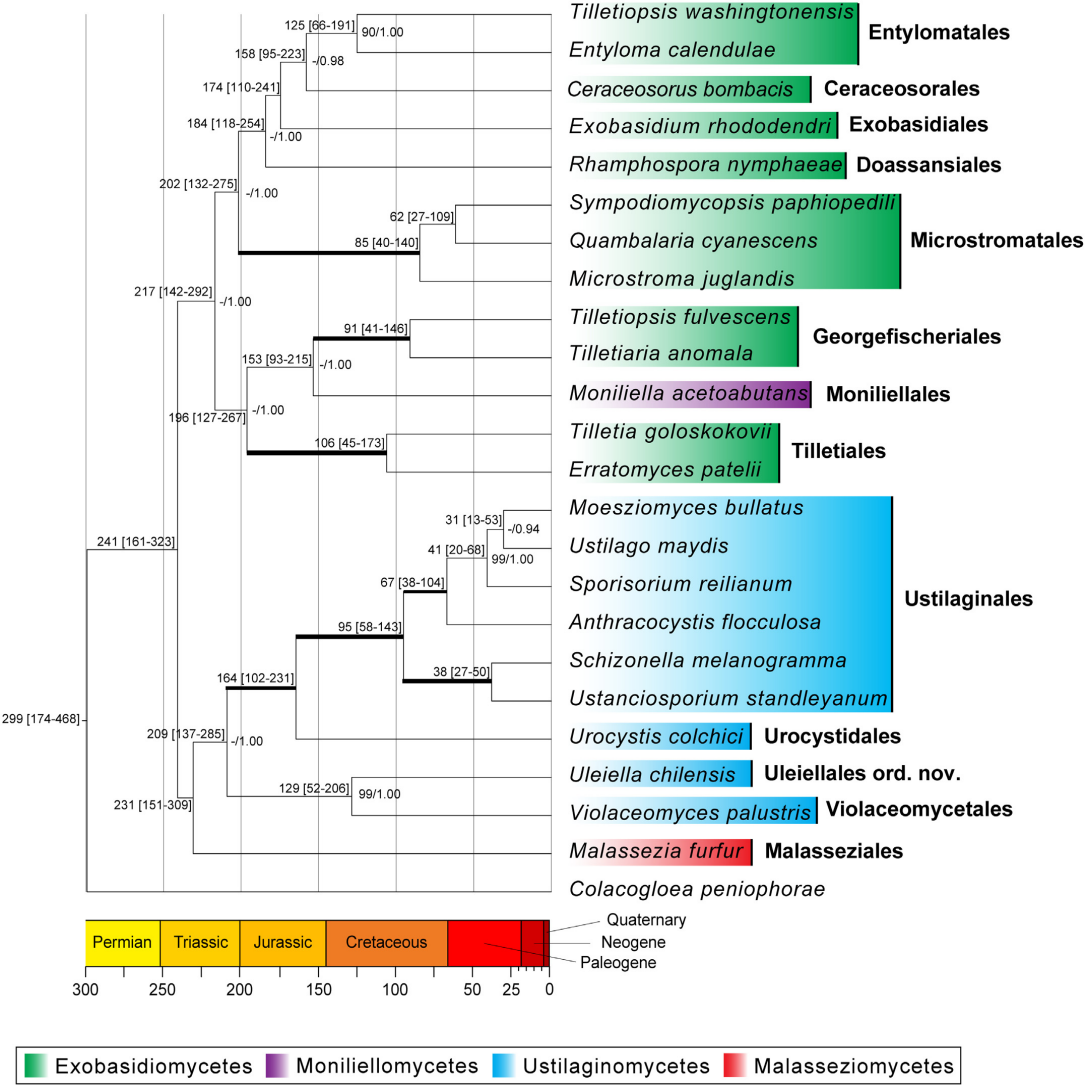
Chronogram for Ustilaginomycotina evolution. The tree topology represents the consensus of trees inferred with BEAST from combined 18S, ITS, 28S, *rpb*2 and *EF1α* sequences from 23 Ustilaginomycotina species and *Colacogloea peniophorae* (Pucciniomycotina) as outgroup. Numbers on branches before slashes are ML bootstrap support (BS) values (≥ 70); numbers on branches after slashes are estimates for *a posteriori* probabilities (PP, ≥ 0.90). The lines in bold indicate a maximum support of 100/1.00. The age estimation values (in million years ago, mya) are given for each node. The age estimation mean is followed by the 95% highest density probability (HDP) range in square brackets. The Ustilaginomycotina classes are depicted (see legend) and they are in agreement with the recently published study by Wang et al. [56].

Divergence time estimates indicate that the stem age of the Ustilaginomycotina is around 450 (293–717) mya (S1 Fig). Within the Ustilaginomycotina, Ustilaginomycetes, Exobasidiomycetes and Malasseziomycetes have a Triassic origin with major order diversifications during the Triassic-Jurassic (~ 250–145 mya). The Uleiellales (*Uleiella chilensis*) described here as new order and Violaceomycetales (*Violaceomyces palustris*) have split relatively recently, approximately 129 (52–206) mya. The Uleiellales/Violaceomycetales share a recent common ancestor with the Urocystidales/Ustilaginales approximately 209 (137–285) mya (Fig 2).

The monophyly of the Ustilaginomycetes and Exobasidiomycetes including the moniliellomyceous *Moniliella acetoabutans* was only supported by Bayesian inference. Malasseziomycetes represented by the yeast *Malassezia furfur* had an isolated and unresolved position (Fig 2). Several deep relationships between Ustilaginomycotina orders were only resolved with significant support values from Bayesian analyses (Fig 2). *Uleiella chilensis* was nested with *Violaceomyces palustris* with significantly high BS (99%) and PP (1.00) support values and a sister to Ustilaginales and Urocystidales (Ustilaginomycetes), supported with a BS value of less than 70 and PP 1.00 (Fig 2). ML and Bayesian analyses of D1/D2 LSU rDNA sequences from a wide taxonomic and phylogenetic spectrum of Ustilaginomycotina recovered congruent phylogenetic trees with relatively lower BS (72%) support and strong PP (95%) support for the monophyly of a group containing *Uleiella chilensis* as sister taxon to the yeasts *Violaceomyces palustris* (Violaceomycetales) and *Tilletiopsis* sp. (DQ404470) (data not shown).

### Sporulation in host tissue and on artificial media

**Fig 3:**
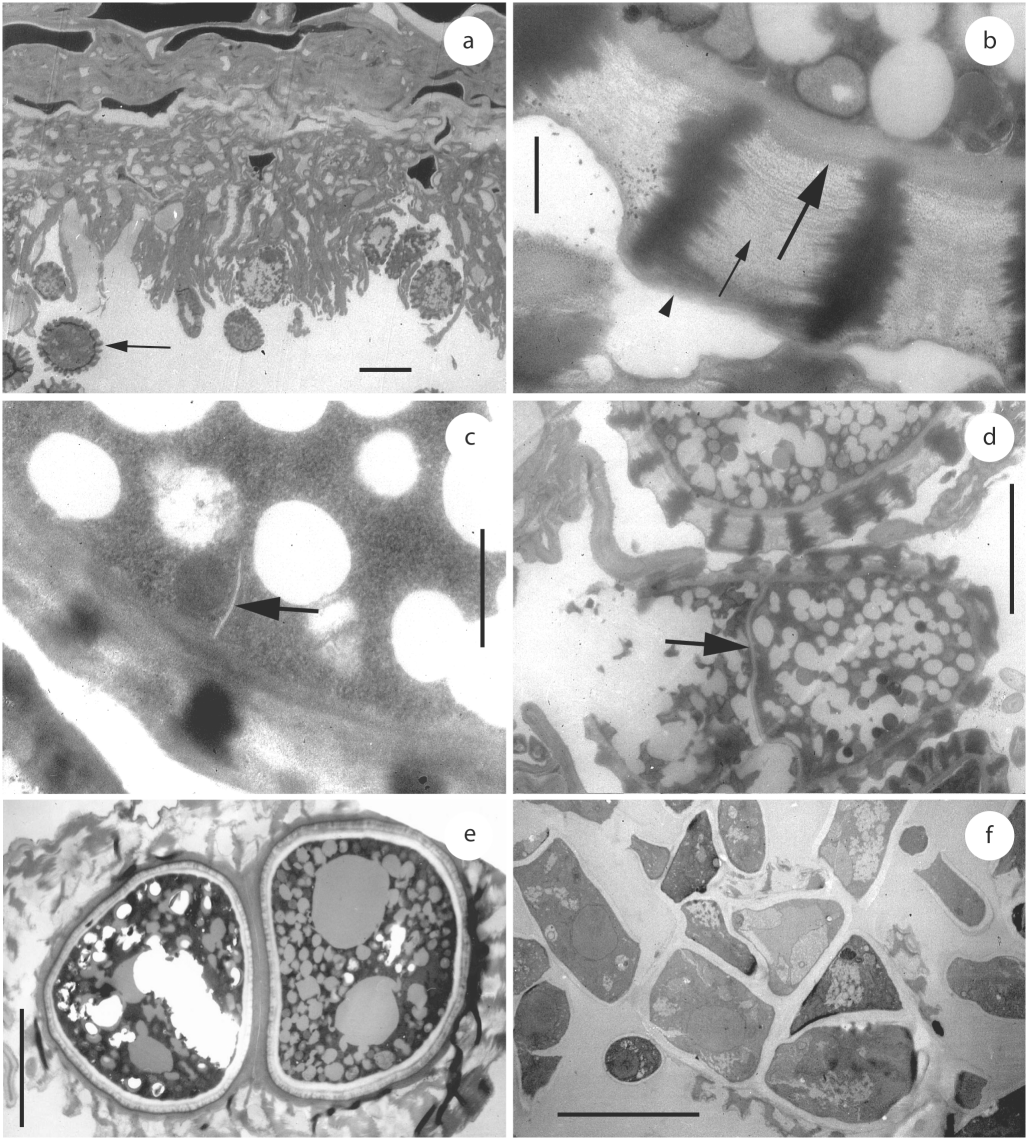
Teliosporogenesis of *Uleiella chilensis* as seen by transmission electron microscopy. Material from a–e was prepared from a herbarium specimen. (a) Section through a sorus showing external teliospores (one is indicated by an arrow). (b) Teliospore wall with a sheath (arrowhead), an exosporium with ornaments (small arrow) and an endosporium (large arrow). (c) Section through a young teliospore with ornaments showing the beginning of septation (arrow). (d) Section through a teliospore showing one complete septum (arrow). (e) Section through a mature teliospore with two more or less rounded segments. (f) Section through a germinating teliospore, showing the multicellular content. Scale bar = 10 μm in (a), 0.2 μm in (b–c) and 0.5 μm in (d–f).

**Fig 4:**
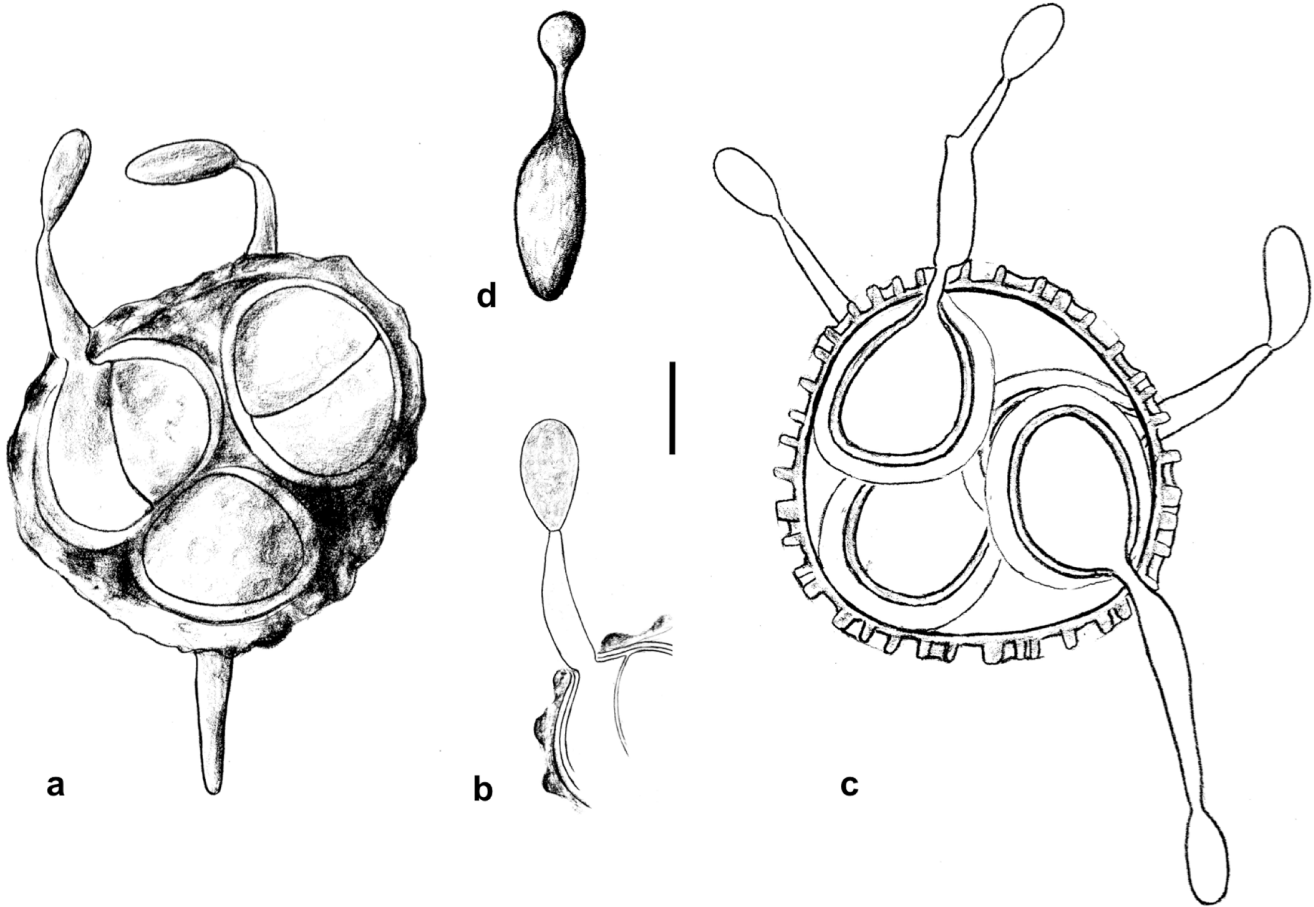
Line drawings of teliospore germination in *Uleiella chilensis*. (a) Teliospore with four segments, three of them visible. Short germination tubes protrude through the primary spore wall and terminate with sporidia (in two cases). (b) Optical section of the left part of a. (c) Optical section of a teliospore with four germination tubes producing terminal sporidia. Wall layers of the primary spore and the internal cells are indicated schematically. (d) Germination of a sporidium with the initial stage of a second sporidium. Scale bar = 5 μm.

**Fig 5:**
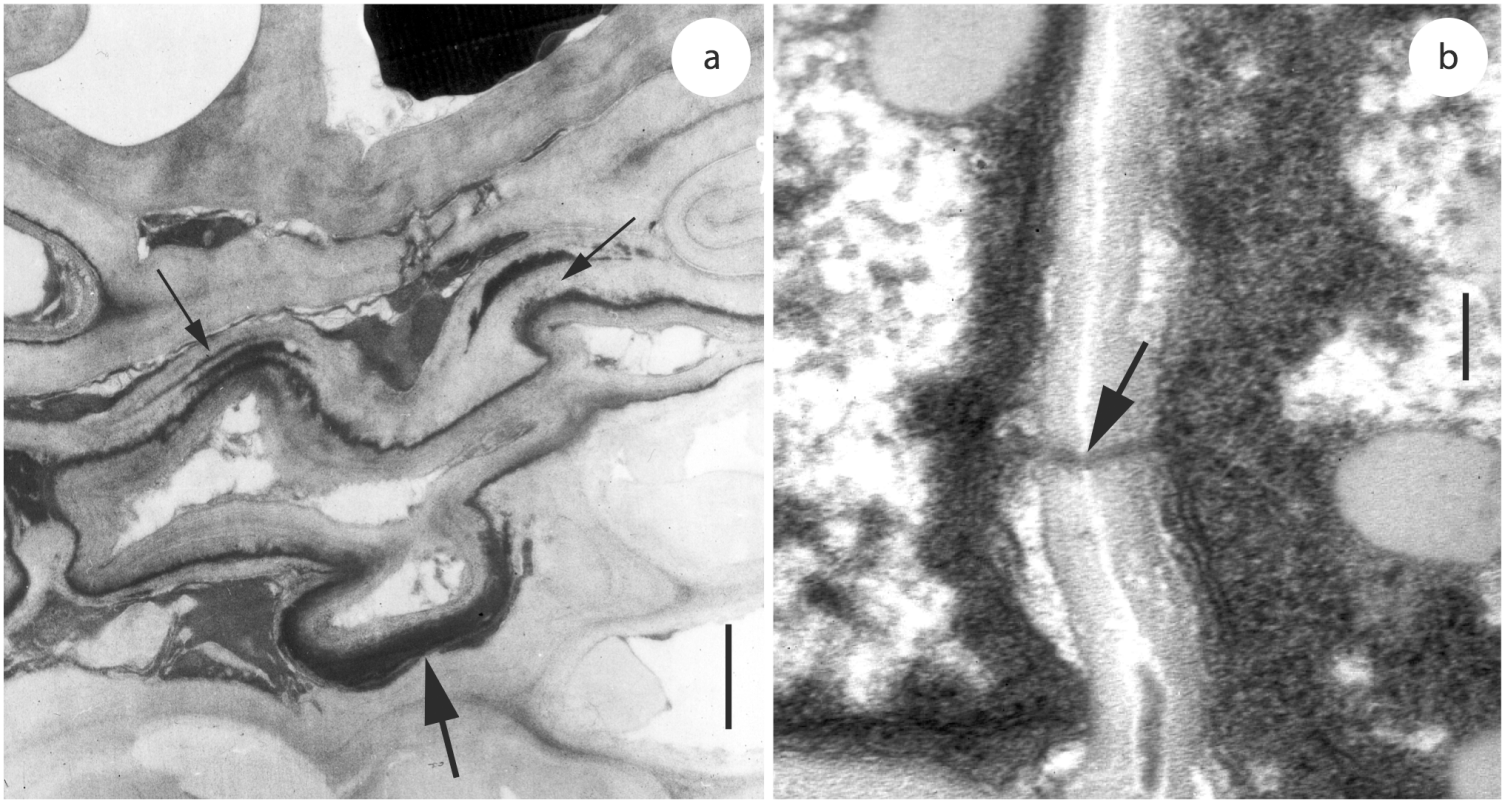
Hyphal characteristics of *Uleiella chilensis* as seen by transmission electron microscopy. Material from (a) was prepared from a herbarium specimen. (a) Section through an intercellular hypha with three short haustorial lobes (arrows). Note the electron-opaque matrix coating the haustorial lobes that appears to have more layers in two of them (small arrows). (b) Section through a hypha showing a plasmodesma-like perforation (arrow). Scale bar = 10 μm in (a) and 0.2 μm in (b).

*Uleiella chilensis* sporulated exclusively on the surface of the host tissue (Figs 1 and 3a). Teliospores were produced singly and their wall consisted of an electron-opaque exosporium with reticulate ornamentation, occasionally embedded and partly covered by remnants of the sheath and the wall of the sporogenous hypha and an electron-transparent more or less two-lamellate endosporium (Fig 3b). During sporogenesis, the teliospores became multi-celled by septation (Fig 3c–3f). Subsequently, the teliospore segments appeared rounded giving the impression of “endospores” (Figs 3e and 4). Soral hyphae were thick-walled and filled the intercellular spaces. Aseptate haustoria arose from intercellular hyphal cells that contacted host cells. Haustoria were not constricted at the penetration point and extended only a short distance into the host cell. Haustoria terminated in the host cell and were surrounded by an electron-opaque matrix (Fig 5a). In the initial stages of interaction, a matrix began to develop and appeared on the host side between the host cell wall and the host cytoplasm. Occasionally, the matrix was arranged in two or more layers, separated from each other by a secondary layer of host origin (Fig 5a). Septa in soral hyphae were rare and thick-walled. Mature septa in soral hyphae and in cultural hyphae were poreless. Central swellings of variable size with an interrupted or branched electron-transparent middle layer or a plasmodesma-like perforation were usually present (Fig 5b).

Teliospore cells germinated after one day on WA and MYP with hyphae on which monokaryotic conidia arose asynchronously (Fig 4). Usually, conidia were produced on short germination tubes. The conidiogenous hyphae became zigzag in profile. Septa and branches developed so that the resulting cultures consisted of more or less pseudohyphae on which masses of conidia arose. On MYP (but not on WA), the formation of conidia stopped after a month, followed by the formation of thick-walled cells in chains. Simultaneously, the colour of the cultures changed from white to dark olivaceous green. Yeasts and ballistoconidia were absent.

## Discussion

The results of the present study estimated smut fungi to have originated in the Ordovician period (~450 mya), which is in agreement with previous studies [42, 43]. Our results indicate that most orders within the Ustilaginomycotina diverged during the Triassic–Jurassic period (Fig 2). Therefore, it appears that the origins of the ancestral lineages (the crown node) of smut fungi date back before the radiation of angiosperms and coincide with the major expansion of gymnosperms [44]. Considering the age estimates that place the origin (the stem node) of smut fungi before the diversification of vascular plants, as well as the lack of resolution in the basal nodes, we propose the following evolutionary history for the Ustilaginomycotina: Early smut fungi must have been living as saprobic organisms as no host plants would have existed at this time. This is also supported by the widely distributed yeast genus *Malassezia*, which diverged early and which is known to occur both as a saprophyte and as a parasite on animals [45]. It therefore seems appropriate to assume that the most recent common ancestor of Uleiellales and Violaceomycetales also had a saprobic lifestyle. The Violaceomycetales subsequently are specialised as endophytes of Pteridophyta or stayed saprobic [46], whereas the Uleiellales changed their lifestyle and became obligate parasites of the *Araucaria* lineage. In the genus *Uleiella*, only two extant species are known, *U*. *chilensis* and *U*. *paradoxa*. Interestingly, the respective hosts, *Araucaria araucana* and *A*. *angustifolia*, are closely related and are the only representatives of the genus in South America. The fact that *Uleiella* is restricted to South America while *Araucaria* species occur disjunct across the whole Southern Hemisphere might be resolved when we assume that the transition in the lifestyle of the Uleiellales happened after the separation of continents (this is also supported by the lower bound of the age estimate for *U*. *chilensis* and *V*. *palustris*). Furthermore, as both species of South American *Araucaria* host a unique species of *Uleiella*, we propose that it was either coevolution between this branch of *Araucaria* and the genus *Uleiella*, or that there was a host shift followed by allopatric speciation as the most likely scenarios explaining the evolutionary history. This evolutionary hypothesis is in agreement with the coevolutionary dynamics between hosts and parasites as postulated by [47].

After the emergence of the angiosperms, the orders in the Ustilaginomycotina that became associated with them underwent rapid diversification. The low diversity of the parasitic smut fungi on gymnosperms may be explained by low diversification and high extinction rates, as well as the geographic isolation of their hosts.

In our multi-gene analysis (Fig 2), *Uleiella chilensis* and *Violaceomyces palustris* clustered together forming a sister clade to the Urocystidales and Ustilaginales (Ustilaginomycetes). The relationship between *Uleiella* and the Urocystidales [1, 2] is supported by the morphology of teliospores, which have a similar appearance compared to those of *Mundkurella* [48]. The lack of pores at the hyphal septa of *Uleiella chilensis* supports a closer relationship to the families Mycosyringaceae and Glomosporiaceae, which belong to the Urocystidales and almost all Ustilaginales (with the exception of Melanotaeniaceae). The presence of enlarged interaction zones [2] supports the placement in the class Ustilaginomycetes *sensu* Begerow *et al*. [49].

Interestingly, the asynchronous development of the conidia in *Uleiella chilensis* indicates that they do not represent basidiospores and, consequently, the teliospore germlings do not represent basidia. However, it is known that in many smut fungi, teliospore germination often depends on environmental conditions, ranging from true holobasidia to septate hyphae that sometimes bear conidia [50, 51]. Possibly, teliospore germination in *Uleiella chilensis* represents an atypical germination resulting from an adaptation to extreme environmental factors. *Araucaria araucana*, the host of *Uleiella chilensis*, occurs in sites that experience extreme temperature and humidity conditions in the Andes and the Chilean coastal range. In order to better understand whether teliospore germination effectively represents an adaptive mechanism, further field investigations are needed. The ecology, the deep genetic divergence and the presence of haustoria and poreless septa characterize *Uleiella* as a unique evolutionary lineage within Ustilaginomycotina for which we describe a new order.

### Taxonomy

**Uleiellales** Garnica, K. Riess, M. Schön, H. Butin, M. Lutz, Oberw. & R. Bauer, ord. nov.

[MycoBank #804545]

Member of Ustilaginomycotina [52] and class Ustilaginomycetes [53] parasitizing gymnosperms, having haustoria and poreless septa. Equivalent to Uleiellaceae [54].

Type genus: *Uleiella* J. Schröt. [55], p. 65, includes two species *U*. *chilensis* Dietel & Neger and *U*. *paradoxa* J. Schröt.

The newly described order is phylogenetically closely related to the order Violaceomycetales, but differs considerably in its ecology. Violaceomycetales includes a single species, *Violaceomyces palustris* that apparently occurs endophytically associated with *Salvinia* ferns from invaded mostly aquatic habitats in Louisiana, USA [46]. As *Violaceomyces palustris* and also *Tilletiopsis* sp. (DQ404470) are known only from their yeast phases and other cellular or subcellular features are unknown it is difficult to carry out morphological comparisons with *Uleiella chilensis*.

## Supporting Information

S1 Data A concatenated alignment of dataset 1 containing the 18S, 28S and RPB1 sequences used for estimating the age of the Basidiomycota (4398 bp in length). For GenBank accession numbers, see S1 Table.
(NEXUS)

S2 Data A concatenated alignment of Dataset 2 containing the 18S, ITS, 28S, *rpb*2 and *EF1α* sequences used for estimating the age of the Ustilaginomycotina (5767 bp in length). For GenBank accession numbers, see S2 Table.
(NEXUS)

S3 Data This file contains information about the priors and parameters used in BEAST to obtain the age estimates of the Basidiomycota (dataset 1). For GenBank accession numbers, see S1 Table.
(XML)

S4 Data This file contains information about the priors and parameters used in BEAST to obtain the age estimates of the Ustilaginomycotina (dataset 2). For GenBank accession numbers, see S2 Table.
(XML)

S1 Fig Chronogram of Basidiomycota evolution inferred from concatenated 18S, 28S and RPB1 sequences. Numbers on branches before slashes are ML bootstrap support values (≥ 70); numbers on branches after slashes are estimates for *a posteriori* probabilities (≥ 0.90). The ascomycetes *Candida albicans*, *Taphrina deformans* and *Saccharomyces cerevisae* were used as outgroup. The lines in bold indicate a maximum support of 100/1.00. The age estimation values (in million years ago, mya) are given for each node. The age estimation mean is followed by the 95% highest density probability (HDP) range in square brackets. Arrows indicate the nodes used for the secondary calibration (dataset 2).
(PDF)

S1 Table Specimens and their corresponding GenBank accession numbers used for age estimations of Basidiomycota (dataset 1). Numbers in bold typeface indicate new sequences from this study.
(XLS)

S2 Table Specimens and their corresponding GenBank accession numbers used for age estimations of Ustilaginomycotina (dataset 2). Numbers in bold typeface indicate new sequences from this study.
(XLS)

## References

[1] Bauer R, Begerow D, Oberwinkler F, Piepenbring M, Berbee ML. Ustilaginomycetes In: McLaughlin DJ, McLaughlin EG, Lemke PA, editors. The Mycota, Volume 7, Systematics and Evolution. Heidelberg: Springer; 2001 pp. 57–83.

[2] Bauer R, Oberwinkler F, Vánky K. Ultrastructural markers and systematics in smut fungi and allied taxa. Can J Bot. 1997;75: 1273–1314.

[3] Bauer R, Oberwinkler F, Vánky K. Ustilaginomycetes on Osmunda. Mycologia. 1999;91: 669–675.

[4] Begerow D, Schäfer AM, Kellner R, Yurkov A, Kemler M, Oberwinkler F, et al Ustilaginomycotina In: McLaughlin DJ, Spatafora JW, editors. The Mycota, Volume 7A, Systematics and Evolution. Heidelberg: Springer; 2014 pp. 295–329.

[5] Begerow D, Bauer R, Boekhout T. Phylogenetic placements of ustilaginomycetous anamorphs as deduced from nuclear LSU rDNA sequences. Mycol Res. 2000;104: 53–60.

[6] Wang Q-M, Theelen B, Groenewald M, Bai F-Y, Boekhout T. Moniliellomycetes and Malasseziomycetes, two new classes in Ustilaginomycotina. Persoonia. 2014;33: 41–47. 10.3767/003158514X682313 25737592 PMC4312936

[7] Bauer R, Vánky K, Begerow D, Oberwinkler F. Ustilaginomycetes on Selaginella. Mycologia. 1999;91: 475–484.

[8] Bauer R, Begerow D, Vanky K, Oberwinkler F. Georgefischeriales: a phylogenetic hypothesis. Mycol Res. 2001;104: 416–424.

[9] Bauer R, Lutz M, Oberwinkler F. Gjaerumia, a new genus in the Georgefischeriales (Ustilaginomycetes). Mycol Res. 2005;109: 1250–1258. 16279418 10.1017/s0953756205003783

[10] Begerow D, Bauer R, Oberwinkler F. *Muribasidiospora*: Microstromatales or Exobasidiales? Mycol Res. 2001;105: 798–810.

[11] Butin H, Peredo HL. Hongos parásitos en coníferas de América del Sur, con especial referencia a Chile. Bibl Mycol. 1986;101: 1–100.

[12] Miller CN. Mesozoic conifers. Bot Rev. 1977;43: 217–280

[13] Setoguchi H, Pintaud JC, Jaffre T, Veillon JM. Phylogenetic relationships within Araucariaceae based on *rbcL* gene sequences. Am J Bot. 1998;85: 1507–1516. 21680310

[14] Stockey RA. The Araucariaceae: an evolutionary perspective. Rev Palaeobot Palynol. 1982;37: 133–154.

[15] Stockey RA, Nishida M, Nishida H. Upper Cretaceous araucarian cones from Hokkaido: Araucaria nihongii sp. nov. Rev Palaeobot Palynol. 1992;72: 27–40.

[16] Kranitz ML, Biffin E, Clark A, Hollingsworth ML, Ruhsam M, Gardner MF, et al Evolutionary Diversification of New Caledonian Araucaria. PLoS ONE. 2014;9: e110308 10.1371/journal.pone.0110308 25340350 PMC4207703

[17] Axsmith BJ, Escapa IH, Huber P. An araucarian conifer bract-scale complex from the lower Jurassic of Massachusetts: implications for estimating phylogenetic and stratigraphic congruence in the Araucariaceae. Palaeontol Electron. 2008;11: 13A.

[18] Matheny PB, Liu YJ, Ammirati JF, Hall BD. Using RPB1 sequences to improve phylogenetic inference among mushrooms. Am J Bot. 2002;89: 688–698. 10.3732/ajb.89.4.688 21665669

[19] Liu YL, Whelen S, Hall BD. Phylogenetic relationships among ascomycetes: evidence from an RNA polymerase II subunit. Mol Phylogenet Evol. 1999;16: 1799–1808. 10.1093/oxfordjournals.molbev.a026092 10605121

[20] Matheny PB. Improving phylogenetic inference of mushrooms with RPB1 and RPB2 nucleotide sequences (Inocybe, Agaricales). Mol Phylogenet Evol. 2005;35: 1–20. 15737578 10.1016/j.ympev.2004.11.014

[21] Rehner SA, Buckley E. A *Beauveria* phylogeny inferred from nuclear ITS and EF1-a sequences: evidence for cryptic diversification and links to *Cordyceps* teleomorphs. Mycologia. 2005;97: 84–98. 16389960 10.3852/mycologia.97.1.84

[22] O’Donnell KL. Fusarium and its near relatives In: Reynolds DR, Taylor JW, editors. The Fungal Holomorph: Mitotic, Meiotic and Pleomorphic Speciation in Fungal Systematics. Wallingford: CAB International; 1993 pp. 225–233.

[23] Hopple JS, Vilgalys R. Phylogenetic relationships in the mushroom genus *Coprinus* and dark-spored allies based on sequence data from the nuclear gene coding for the large ribosomal subunit RNA: divergent domains, outgroups, and monophyly. Mol Phylogenet Evol. 1999;13: 1–19. 10508535 10.1006/mpev.1999.0634

[24] Riess K, Oberwinkler F, Bauer R, Garnica S. High genetic diversity at the regional scale and possible speciation in *Sebacina epigaea* and *S*. *incrustans*. BMC Evol Biol. 2013;13: 102 10.1186/1471-2148-13-102 23697379 PMC3665632

[25] Liu YJ, Hudson MC, Hall BD. Loss of the flagellum happened only once in the fungal lineage: phylogenetic structure of Kingdom Fungi inferred from RNA polymerase II subunit genes. BMC Evol Biol. 2006;6: 74 17010206 10.1186/1471-2148-6-74 PMC1599754

[26] Garnica S, Weiß M, Oertel B, Ammirati J, Oberwinkler F. Phylogenetic relationships in *Cortinarius*, section *Calochroi*, inferred from nuclear DNA sequences. BMC Evol Biol. 2009;9: 1 10.1186/1471-2148-9-1 19121213 PMC2653478

[27] Vilgalys R, Hester M. Rapid genetic identification and mapping of enzymatically amplified ribosomal DNA from several *Cryptococcus* species. J Bacteriol. 1990;172: 4238–4246. 2376561 10.1128/jb.172.8.4238-4246.1990 PMC213247

[28] Katoh K, Kuma K, Toh H, Miyata T. MAFFT version 5: improvement in accuracy of multiple sequence alignment. Nucleic Acids Res. 2005;33: 511–518. 15661851 10.1093/nar/gki198 PMC548345

[29] Katoh K, Toh H. Recent developments in the MAFFT multiple sequence alignment program. Brief Bioinform. 2008;9: 286–298. 10.1093/bib/bbn013 18372315

[30] Capella-Gutiérrez S, Silla-Martínez JM, Gabaldón T. trimAl: a tool for automated alignment trimming in large-scale phylogenetic analyses. Bioinformatics. 2009;25: 972–1973. 10.1093/bioinformatics/btp348 PMC2712344 19505945

[31] Subramanian AR, Kaufmann M, Morgenstern B. DIALIGN-TX: greedy and progressive approaches for segment-based multiple sequence alignment. Algorithm Mol Biol. 2008;3: 6. 10.1186/1748-7188-3-6 PMC2430965 18505568

[32] Rambaut A. Se-Al Sequence Alignment Editor v.2.0a11 Carbon. 2002. Available: http:// tree.bio.ed.ac.uk/software/seal

[33] Stamatakis A. RAxML version 8: a tool for phylogenetic analysis and post-analysis of large phylogenies. Bioinformatics. 2014;30: 1312–1313. 10.1093/bioinformatics/btu033 24451623 PMC3998144

[34] Felsenstein J. Confidence limits on phylogenies: an approach using the bootstrap. Evolution. 1985;39: 783–791. 28561359 10.1111/j.1558-5646.1985.tb00420.x

[35] Ronquist F, Teslenko M, van der Mark P, Ayres DL, Darling A, Höhna S, et al MrBayes 3.2: efficient Bayesian phylogenetic inference and model choice across a large model space. Syst Biol. 2012;61: 539–542. 10.1093/sysbio/sys029 22357727 PMC3329765

[36] Drummond AJ, Suchard MA, Xie D, Rambaut A. Bayesian phylogenetics with BEAUti and the BEAST 1.7 Mol Biol Evol. 2012;29: 1969–1973. 10.1093/molbev/mss075 22367748 PMC3408070

[37] LePage BA, Currah RS, Stockey RA, Rothwell GW. Fossil ectomycorrhizae from the Middle Eocene. Am J Bot. 1997;84: 410–412. 21708594

[38] Hibbett DS, Grimaldi D, Donoghue MJ. Fossil mushrooms from Cretaceous and Miocene ambers and the evolution of homobasidiomycetes. Am J Bot. 1997;84: 981–991. 21708653

[39] Rambaut A, Suchard MA, Xie D, Drummond AJ. Tracer v.1.6. 2014. Available: http://beast.bio.ed.ac.uk/Tracer.

[40] Forest F. Calibrating the tree of life: Fossils, molecules and evolutionary timescales. Ann Botany. 2009;104: 789–794. 19666901 10.1093/aob/mcp192 PMC2749537

[41] Piepenbring M, Bauer R, Oberwinkler F. Teliospores of smut fungi. Teliospore walls and the development of ornamentation studied by electron microscopy. Protoplasma. 1998;204: 155–169.

[42] Floudas D, Binder M, Riley R, Barry K, Blanchette RA, Henrissat B, et al The Paleozoic origin of enzymatic lignin descomposition reconstructed from 31 fungal genomes. Science. 2012;336: 1715–1719. 10.1126/science.1221748 22745431

[43] Taylor JW, Berbee ML. Dating divergences in the Fungal Tree of Life: review and new analyses. Mycologia. 2006;98: 838–849. 17486961 10.3852/mycologia.98.6.838

[44] Willis KJ, McElwain JC. The evolution of plants. New York: Oxford University Press; 2002.

[45] Amend A. From Dandruff to Deep-Sea Vents: Malassezia-like Fungi Are Ecologically Hyper-diverse. PLoS Pathog. 10; 20148: e1004277. 25144294 10.1371/journal.ppat.1004277 PMC4140847

[46] Albus S, Toome M, Aime MC. *Violaceomyces palustris* gen. et sp. nov. and a new monotypic lineage,Violaceomycetales ord. nov. in Ustilaginomycetes. Mycologia 2015; 10.3852/14-260 26297779

[47] de Vienne DM, Refrégier G, López-Villavicencio M, Tellier A, Hood ME, Giraud T. Cospeciation vs host-shift speciation: methods for testing, evidence from natural associations and relation to coevolution. New Phytol. 2013;198: 347–385. 10.1111/nph.12150 23437795

[48] Vánky K. Illustrated Genera of Smut Fungi. 2nd ed. St. Paul: American Phytopathological Society Press; 2002.

[49] Begerow D, Stoll M, Bauer R. A phylogenetic hypothesis of Ustilaginomycotina based on multiple gene analyses and morphological data. Mycologia. 2006;98: 906–916. 17486967 10.3852/mycologia.98.6.906

[50] Ingold CT. Aerial sporidia of *Ustilago hypodytes* and *Sorosporium saponariae*. Trans Br Mycol Soc. 1987;89: 471–475.

[51] Piepenbring M, Bauer R. Noteworthy germinations of some Costa Rican Ustilaginales. Mycol Res. 1995;99: 853–858.

[52] Bauer R, Begerow D, Sampaio JP, Weiß M, Oberwinkler F. The simple-septate basidiomycetes: a synopsis. Mycol Prog. 2006;5: 41–66.

[53] Vánky K. The emended Ustilaginaceae of the modern classificatory system for smut fungi. Fungal Divers. 2001;6: 131–147.

[54] Hibbett DS, Binder M, Bischoff JF, Blackwell M, Cannon PF, Eriksson OE, et al A higher-level phylogenetic classification of the Fungi. Mycol Res. 2007;11: 509–47. 10.1016/j.mycres.2007.03.004 17572334

[55] Schröter J. Uleiella gen. nov. Hedwigia Beibl. 1894;33: 64–66.

[56] Wang QM, Begerow D, Groenewald M, Liu XZ, Theelen B, Bai FY Boekhout T. Multigene phylogeny and taxonomic revision of yeasts and related fungi in the Ustilaginomycotina. Stud. Mycol. 2015;81: 55–83. 26955198 10.1016/j.simyco.2015.10.004 PMC4777779

