# Correction: On the Evolutionary History of *Uleiella chilensis*, a Smut Fungus Parasite of *Araucaria araucana* in South America: Uleiellales ord. nov. in Ustilaginomycetes

**DOI:** 10.1371/journal.pone.0152646

**Published:** 2016-03-24

**Authors:** Kai Riess, Max E. Schön, Matthias Lutz, Heinz Butin, Franz Oberwinkler, Sigisfredo Garnica

There are errors in the Funding section. The correct funding information is as follows: This research was funded by the German Foundation Research (OB 24/30-1) and the Open Access Publishing Fund of Tuebingen University.
