# Supplementary figures and images for: On the Evolutionary History of Uleiella chilensis, a Smut Fungus Parasite of Araucaria araucana in South America: Uleiellales ord. nov. in Ustilaginomycetes
Source: PLoS One. 2016 Jan 20;11(1):e0147107. doi: 10.1371/journal.pone.0147107 (PMC4738420; doi:10.1371/journal.pone.0147107)

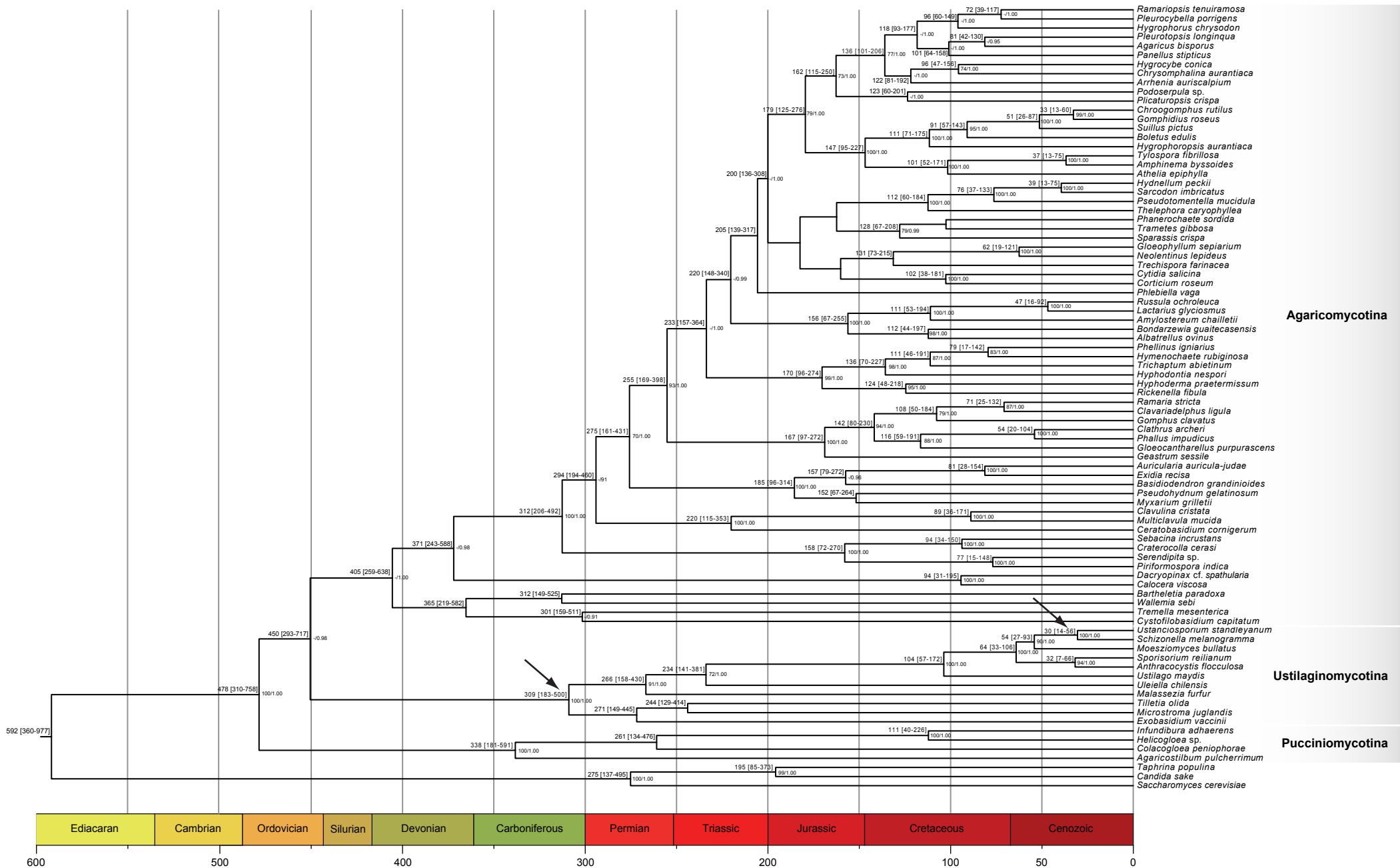

Supplement: S1 Fig — Numbers on branches before slashes are ML bootstrap support values (≥ 70); numbers on branches after slashes are estimates for a posteriori probabilities (≥ 0.90). The ascomycetes Candida albicans, Taphrina deformans and Saccharomyces cerevisae were used as outgroup. The lines in bold indicate a maximum support of 100/1.00. The age estimation values (in million years ago, mya) are given for each node. The age estimation mean is followed by the 95% highest density probability (HDP) range in square brackets. Arrows indicate the nodes used for the secondary calibration (dataset 2). (PDF) [file pone.0147107.s005.pdf]
